# Insights From the SmokeFree.gov Initiative Regarding the Use of Smoking Cessation Digital Platforms During the COVID-19 Pandemic: Cross-sectional Trends Analysis Study

**DOI:** 10.2196/24593

**Published:** 2021-03-22

**Authors:** Sherine El-Toukhy

**Affiliations:** 1 Division of Intramural Research National Institute on Minority Health & Health Disparities National Institutes of Health Bethesda, MD United States

**Keywords:** COVID-19, smoking, cessation, mHealth, risk, digital platform, social distancing, lockdown, trend

## Abstract

**Background:**

Smoking is a plausible risk factor for COVID-19 progression and complications. Smoking cessation digital platforms transcend pandemic-driven social distancing and lockdown measures in terms of assisting smokers in their quit attempts.

**Objective:**

This study aims to examine trends in the number of visitors, followers, and subscribers on smoking cessation digital platforms from January to April 2020 and to compare these traffic data to those observed during the same 4-month period in 2019. The examination of prepandemic and postpandemic trends in smoking cessation digital platform traffic can reveal whether interest in smoking cessation among smokers is attributable to the COVID-19 pandemic.

**Methods:**

We obtained cross-sectional data from daily visitors on the SmokeFree website; the followers of six SmokeFree social media accounts; and subscribers to the SmokeFree SMS text messaging and mobile app interventions of the National Cancer Institute’s SmokeFree.gov initiative platforms, which are publicly available to US smokers. Average daily percentage changes (ADPCs) were used to measure trends for the entire 2020 and 2019 study periods, whereas daily percentage changes (DPCs) were used to measure trends for each time segment of change within each 4-month period. Data analysis was conducted in May and June 2020.

**Results:**

The number of new daily visitors on the SmokeFree website (between days 39 and 44: DPC=18.79%; 95% CI 5.16% to 34.19%) and subscribers to the adult-focused interventions QuitGuide (between days 11 and 62: DPC=1.11%; 95% CI 0.80% to 1.43%) and SmokeFreeTXT (between days 11 and 89: DPC=0.23%; 95% CI 0.004% to 0.47%) increased, but this was followed by declines in traffic. No comparable peaks were observed in 2019. The number of new daily subscribers to quitSTART (ie, the teen-focused intervention) trended downward in 2020 (ADPC=−1.02%; 95% CI −1.88% to −0.15%), whereas the overall trend in the number of subscribers in 2019 was insignificant (*P*=.07). The number of SmokeFree social media account followers steadily increased by <0.1% over the 4-month study periods in 2019 and 2020.

**Conclusions:**

Peaks in traffic on the SmokeFree website and adult-focused intervention platforms in 2020 could be attributed to an increased interest in smoking cessation among smokers during the COVID-19 pandemic. Coordinated campaigns, especially those for adolescents, should emphasize the importance of smoking cessation as a preventive measure against SARS-CoV-2 infection and raise awareness of digital smoking cessation platforms to capitalize on smokers’ heightened interest during the pandemic.

## Introduction

The novel SARS-CoV-2 causes COVID-19. Initially, COVID-19 was identified in December 2019 from a cluster of pneumonia cases of unknown causes in Wuhan, China [1]. COVID-19 has spread throughout the globe and has claimed hundreds of thousands of lives. In the United States, the official number of confirmed cases has surpassed 20 million, and 345,000 fatalities have been reported as of December 31, 2020 [2].

As researchers have attempted to identify risk factors that are associated with SARS-CoV-2 infection and COVID-19–related complications, smoking has emerged as a plausible candidate [3]. Smoking is detrimental to health. Therefore, smokers are more susceptible to the development of disease (specifically pulmonary illnesses) and poor symptoms and outcomes (ie, those associated with health conditions such as asthma and obesity) [4]. Since COVID-19 is primarily a respiratory illness, it is important to note that smoking-induced pathophysiological changes can result in weakened immune responses, inflammation marker development, genetic changes in lung tissue, structural changes in the respiratory tract, and the dysfunction of the lungs. Therefore, smokers are more susceptible to the onset of respiratory illnesses [5,6]. Indeed, studies have shown that smokers have a higher risk of bacterial and viral infection (eg, invasive pneumococcal infection, influenza infection, and infection from coronaviruses like Middle East respiratory syndrome–related coronavirus) than nonsmokers [6,7]. Systematic reviews and meta-analytic evidence have shown that smoking is a risk factor for coronavirus infection, severity, and mortality [8-12]. However, evidence has been inconclusive and contradictory [13,14].

Smoking cessation is an advisable preventive measure against SARS-CoV-2 infection [15]. Compared to nonsmokers, smokers and ex-smokers have significantly higher stress levels due to their susceptibility to SARS-CoV-2 infection and COVID-19 severity [16], which are known health belief model constructs that affect behavioral intentions and behaviors [17]. The COVID-19 pandemic can be considered a naturally occurring cueing event that has motivated smokers to engage in preventive behaviors for reducing their risk of contracting COVID-19 [18]. For example, studies have shown that receiving a new disease diagnosis is associated with quitting smoking [19,20]. Indeed, public health professionals have advised smokers to use interventions that are proven to be effective in helping smokers quit smoking, which can reduce their risk of SARS-CoV-2 infection and COVID-19–related complications [21-23].

As a result of the lockdown and physical distancing measures that have been implemented to reduce the transmission of SARS-CoV-2, digital platforms for smoking cessation resources and interventions have superseded in-person cessation support services [24,25]. Web searches and social media postings provide insights into public opinions about health issues and opportunities for surveilling disease outbreaks and symptoms [26,27]. Furthermore, digital interventions have proven to be effective in inducing behavioral changes, such as quitting smoking [28]. With regard to COVID-19, a study on Google Trends reported that there was no increase in the incidence of smoking cessation–related search terms (eg, “smoking cessation,” “nicotine gum,” and “quit smoking”) from January to April 2020 [29]. Furthermore, hashtags such as #Quit4COVID are prominent on Twitter [30], and researchers have used Facebook data to index social connectedness data that correlate with the geographic spread of COVID-19 [31]. Preliminary evidence from the United Kingdom has also shown that there has been no increase in the number of downloads for Smoke Free (ie, a smoking cessation mobile app) as a result of the COVID-19 outbreak [32].

Traffic on smoking cessation digital platforms is a proxy measure of smokers’ interest in smoking cessation and quit attempts during the COVID-19 pandemic. One such suite of digital platforms that is freely available to smokers in the United States is the National Cancer Institute’s SmokeFree.gov initiative (SFGI) [33,34]. Initially, the SFGI had a single website that launched in 2003 [35]. The SFGI now has multiple platforms, including the main SFGI website, 6 social media accounts, 2 mobile apps (ie, QuitGuide and quitSTART), and 6 SMS text messaging interventions. Around 7-8 million smokers use SFGI platforms every year, and these platforms have an estimated efficacy that ranges from approximately 10% to 30% [35]. The SmokeFree website hosts information on all SFGI smoking cessation programs. SFGI programs typically appear as the top search results on search engines. They are also featured on the webpages of authorities on tobacco control, such as the Centers for Disease Control and Prevention [36] and the Food and Drug administration [37]. SmokeFree SMS text messaging and mobile app interventions for smoking cessation are based on social behavior theory; preliminary studies have shown the acceptability and effectiveness of several SFGI programs [38,39]. The aims of this study are (1) to characterize daily trends in traffic on SFGI digital platforms over 4 months (ie, from January 1 to April 30, 2020); and (2) to compare these trends to those from January to April 2019. We hypothesized that traffic on SFGI digital platforms would increase over the course of the pandemic and that these trends would be qualitatively different from prepandemic trends (ie, those observed in 2019).

## Methods

Data were obtained from the National Cancer Institute’s SmokeFree smoking cessation digital platforms [33]. We obtained aggregated data on the number of new daily visitors on the SmokeFree website, the number of followers on six social media platforms on each given day (ie, SmokeFree Veterans Facebook, SmokeFree Women Facebook, SmokeFreeUS Facebook, SmokeFreeUS Instagram, SmokeFreeUS Pinterest, and SmokeFreeUS Twitter), and the number of new daily subscribers to three smoking cessation interventions (ie, the quitSTART mobile app, QuitGuide mobile app, and SmokeFreeTXT). The study period was January to April 2020. We also obtained data from January to April 2019. Data analysis was conducted in May and June 2020. Since the data were deidentified, approval from an institutional review board was not required.

A joinpoint regression analysis [40,41] was used to model trends in the daily traffic on SmokeFree digital platforms over 4 months and any significant changes in individual time segments within the 4-month period. Joinpoint software (National Cancer Institute) has been used to characterize trends in cancer morbidity, cancer mortality, and the prevalence of risky and healthy behaviors. Joinpoint connects several different line segments with different slopes on a log scale at “joinpoints” and identifies the number and time points in which the trend significantly changes. Joinpoint regression is particularly flexible in terms of modeling nonlinear trends with nontraditional curves, including trends with abrupt changes. Such trends may be present in the traffic on digital platforms for smoking cessation during a pandemic.

Joinpoint analysis involves the calculation of annual percent change and average annual percent change values. However, for this study, time was measured in days. Accordingly, the terms “daily percent change” (DPC) and “average daily percent change” (ADPC) were used (a deviation from joinpoint terminology) to reflect DPCs and ADPCs in the number of daily visitors, followers, or subscribers on each digital platform. The DPC characterizes the trend for each time segment in the joinpoint models. The ADPC is a summary measure of the trend for a prespecified fixed interval; it yields a single number that describes the ADPC for a period of time. For this study, ADPC was estimated for the entire 121- and 120-day periods of interest (ie, from January 1 to April 30, 2020 and 2019, respectively). When the ADPC lies within a single joinpoint segment, the ADPC is equal to the DPC for that segment.

For each platform, we specified a joinpoint model with a minimum of 0 joinpoints and a maximum of 5 joinpoints, as per the recommendations for models with ≥27 data points [42]. We arranged to have at least 5 observations from a joinpoint to either end of the data, excluding the first or last joinpoint if it falls on an observation data point. Further, we arranged to have at least 4 observations between two joinpoints, excluding any joinpoint if it falls on an observation data point. We used the grid search method and Bayesian Information Criteria 3 method to identify the best fitting models and select the final model (Multimedia Appendix 1).

This study only involved the use of deidentified data. Therefore, this study is considered “not human subjects research” and does not require institutional review board review or approval, as per the National Institutes of Health policy and section 45, part 46 of the Code of Federal Regulations. Furthermore, since this study is considered “not human subjects research,” informed consent is not required.

## Results

### SmokeFree Website and Social Media Platforms

#### SmokeFree Website

**Figure 1:**
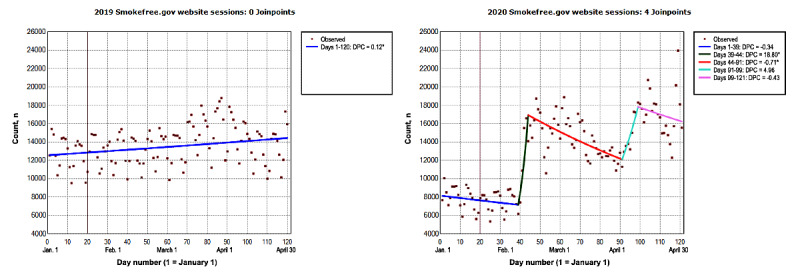
The number of new SmokeFree.gov website visitors from January to April 2019 and 2020. The vertical line represents January 20. On this day in 2020, the first laboratory-confirmed COVID-19 case was identified in the United States. This was reported to the Centers for Disease Control and Prevention on January 22, 2020 [43]. Data on model selection appear in Multimedia Appendix 1. DPC: daily percent change. *The DPC is significantly different from 0 at an α level of .05.

From January to April 2020, the number of new daily visitors on the SmokeFree website ranged from 5344 to 23,959 (Figure 1). The number of new daily visitors increased by a DPC of 18.79% (95% CI 5.16% to 34.19%) during days 39-44, followed by a slight decrease of <1% (DPC=−0.71%; 95% CI −1.01% to −0.41%) during days 44-91 (Table 1). However, the overall trend from January to April was not significant (ADPC=0.57%; 95% CI −0.07% to 1.23%; *P*=.08; Table 2). In 2019, the number of new daily visitors on the SmokeFree website ranged from 9516 to 18,807. The number of new daily visitors on the website steadily increased by 0.11% (95% CI 0.03% to 0.19%).

**Table 1:** Daily percent changes (DPCs) in the number of SmokeFree.gov initiative digital platform visitors, followers, and subscribers from January to April 2019 and 2020.

Digital platform/intervention and time segment		2019^a^				2020^b^		
		Start day	End day	DPC (95% CI)	Start day		End day	DPC (95% CI)
**SmokeFree website**								
	1	1	120	0.116^c^ (0.035 to 0.197)	1		39	−0.336 (−0.737 to 0.066)
	2	N/A^d^	N/A	N/A	39		44	18.796^c^ (5.166 to 34.192)
	3	N/A	N/A	N/A	44		91	−0.712^c^ (−1.012 to −0.411)
	4	N/A	N/A	N/A	91		99	4.959 (−0.308 to 10.506)
	5	N/A	N/A	N/A	99		121	−0.425 (−1.333 to 0.490)
**SmokeFree Veterans Facebook**								
	1	1	17	0.047^c^ (0.036 to 0.058)	1		21	0.038^c^ (0.029 to 0.046)
	2	17	22	−0.064 (−0.156 to 0.027)	21		26	0.178^c^ (0.081 to 0.274)
	3	22	45	0.026^c^ (0.020 to 0.033)	26		99	0.021^c^ (0.020 to 0.022)
	4	45	57	−0.022^c^ (−0.042 to −0.003)	99		116	0.097^c^ (0.086 to 0.109)
	5	57	69	0.072^c^ (0.052 to 0.092)	116		121	−0.043 (−0.111 to 0.025)
	6	69	120	0.023^c^ (0.021 to 0.025)	N/A		N/A	N/A
**SmokeFree Women Facebook**								
	1	1	30	0.014^c^ (0.014 to 0.015)	1		7	0.087^c^ (0.079 to 0.096)
	2	30	52	0.010^c^ (0.009 to 0.010)	7		14	0.050^c^ (0.041 to 0.058)
	3	52	67	0.006^c^ (0.005 to 0.008)	14		73	0.038^c^ (0.038 to 0.038)
	4	67	104	0.017^c^ (0.016 to 0.017)	73		98	0.024^c^ (0.023 to 0.025)
	5	104	111	0.005^c^ (0.00009 to 0.011)	98		107	0.040^c^ (0.035 to 0.046)
	6	111	120	0.024^c^ (0.021 to 0.027)	107		121	0.021^c^ (0.019 to 0.024)
**SmokeFreeUS Facebook**								
	1	1	9	0.058^c^ (0.051 to 0.065)	1		6	0.083^c^ (0.065 to 0.102)
	2	9	23	0.038^c^ (0.034 to 0.041)	6		30	0.038^c^ (0.036 to 0.040)
	3	23	51	0.022^c^ (0.021 to 0.023)	30		36	0.019^c^ (0.001 to 0.038)
	4	51	95	0.034^c^ (0.033 to 0.034)	36		62	0.044^c^ (0.042 to 0.046)
	5	95	100	0.525^c^ (0.031 to 0.073)	62		89	0.024^c^ (0.022 to 0.025)
	6	100	120	0.036^c^ (0.034 to 0.037)	89		121	0.044^c^ (0.043 to 0.045)
**SmokeFreeUS Instagram**								
	1	9^e^	17	0.096^c^ (0.072 to 0.119)	1		52	0.054^c^ (0.052 to 0.055)
	2	17	48	0.057^c^ (0.054 to 0.060)	52		69	0.141^c^ (0.132 to 0.150)
	3	48	70	0.032^c^ (0.027 to 0.038)	69		75	0.049 (−0.001 to 0.099)
	4	70	89	0.053^c^ (0.046 to 0.060)	75		108	0.122^c^ (0.119 to 0.125)
	5	89	105	0.096^c^ (0.086 to 0.105)	108		121	0.088^c^ (0.076 to 0.100)
	6	105	120	0.033^c^ (0.023 to 0.042)	N/A		N/A	N/A
**SmokeFreeUS Pinterest**								
	1	1	23	0.139^c^ (0.128 to 0.149)	1		8	0.064^c^ (0.040 to 0.087)
	2	23	31	−0.002 (−0.058 to 0.054)	8		59	0.017^c^ (0.016 to 0.019)
	3	31	51	0.104^c^ (0.092 to 0.117)	59		72	0.061^c^ (0.050 to 0.071)
	4	51	56	0.222^c^ (0.087 to 0.356)	72		86	0.006 (−0.003 to 0.015)
	5	56	93	0.094^c^ (0.089 to 0.099)	86		94	0.065^c^ (0.042 to 0.089)
	6	93	120	0.044^c^ (0.037 to 0.052)	94		121	0.025^c^ (0.022 to 0.028)
**SmokeFreeUS Twitter**								
	1	1	27	0.001^c^ (0.0009 to 0.002)	1		24	0.008^c^ (0.007 to 0.009)
	2	27	32	−0.033^c^ (−0.046 to −0.020)	24		68	0.002^c^ (0.001 to 0.002)
	3	32	47	−0.0003 (−0.002 to 0.001)	68		86	−0.007^c^ (−0.009 to −0.005)
	4	47	120	0.005^c^ (0.005 to 0.005)	86		100	0.006^c^ (0.004 to 0.009)
	5	N/A	N/A	N/A	100		112	−0.009^c^ (−0.013 to −0.006)
	6	N/A	N/A	N/A	112		121	0.014^c^ (0.009 to 0.019)
**quitSTART**								
	1	1	73	−0.333^c^ (−0.540 to −0.126)	1		11	−8.825^c^ (−12.825 to −4.641)
	2	73	120	0.969^c^ (0.572 to 1.368)	11		79	−0.223 (−0.480 to 0.033)
	3	N/A	N/A	N/A	79		84	12.487 (−6.252 to 34.972)
	4	N/A	N/A	N/A	84		121	−2.000^c^ (−2.613 to −1.383)
**QuitGuide**								
	1	1	25	−2.100^c^ (−3.103 to −1.085)	1		11	−7.457^c^ (−10.641 to −4.158)
	2	25	120	0.034 (-0.096 to 0.165)	11		62	1.118^c^ (0.803 to 1.434)
	3	N/A	N/A	N/A	62		80	−3.145^c^ (−4.658 to −1.607)
	4	N/A	N/A	N/A	80		85	10.136 (−4.467 to 26.972)
	5	N/A	N/A	N/A	85		121	−0.338 (−0.845, 0.171)
**SmokeFreeTXT**								
	1	1	120	0.004 (−0.142 to 0.150)	1		11	−5.411^c^ (−10.059 to −0.523)
	2	N/A	N/A	N/A	11		89	0.235^c^ (0.0004 to 0.470)
	3	N/A	N/A	N/A	89		94	13.050 (−7.871 to 38.724)
	4	N/A	N/A	N/A	94		121	−1.993^c^ (−3.095 to −0.879)

^a^In 2019, January includes days 1-31, February includes days 32-59, March includes days 60-90, and April includes days 91-120.

^b^In 2020, January includes days 1-31, February includes days 32-60, March includes days 61-91, and April includes days 92-121.

^c^The DPC is significantly different from 0 at an α level of .05.

^d^N/A: not applicable.

^e^Data on the number SmokeFree Instagram followers were missing for the first 8 days in January 2019.

**Table 2:** Average daily percent changes (ADPCs) in the number of SmokeFree.gov initiative digital platform visitors, followers, and subscribers from January to April 2019 and 2020.

Digital platform/intervention	ADPC (95% CI) in 2019	ADPC (95% CI) in 2020
SmokeFree website	0.116^a^ (0.035 to 0.197)	0.577 (−0.075 to 1.234)
SmokeFree Veterans Facebook	0.023^a^ (0.018 to 0.028)	0.038^a^ (0.033 to 0.044)
SmokeFree Women Facebook	0.013^a^ (0.013 to 0.014)	0.037^a^ (0.036 to 0.037)
SmokeFreeUS Facebook	0.034^a^ (0.033 to 0.035)	0.039^a^ (0.037 to 0.040)
SmokeFreeUS Instagram	0.056^a^ (0.053 to 0.059)	0.088^a^ (0.085 to 0.091)
SmokeFreeUS Pinterest	0.092^a^ (0.084 to 0.099)	0.028^a^ (0.026 to 0.031)
SmokeFreeUS Twitter	0.002^a^ (0.001 to 0.002)	0.002^a^ (0.001 to 0.002)
quitSTART	0.179 (−0.019 to 0.377)	−1.024^a^ (−1.882 to −0.159)
QuitGuide	−0.399^a^ (−0.628 to −0.170)	−0.351 (−1.068 to 0.370)
SmokeFreeTXT	0.004 (−0.142 to 0.150)	−0.252 (−1.229 to 0.734)

^a^Indicates that the ADPC is significantly different from 0 at an α level of .05.

#### Social Media Platforms

**Figure 2:**
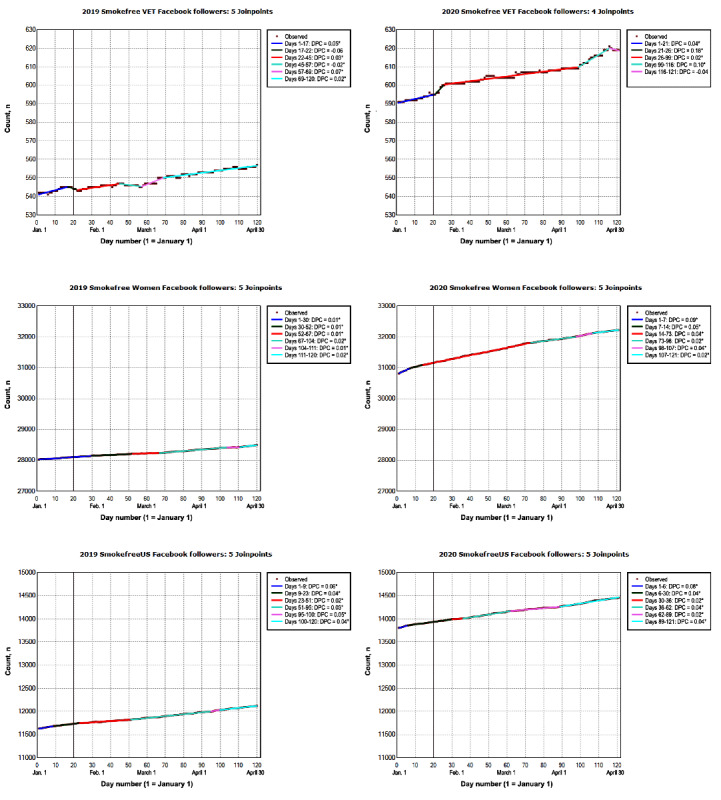
The number of SmokeFree VET Facebook, SmokeFree Women Facebook, and SmokeFreeUS Facebook followers from January to April 2019 and 2020. The vertical line represents January 20. On this day in 2020, the first laboratory-confirmed COVID-19 case was identified in the United States. This was reported to the Centers for Disease Control and Prevention on January 22, 2020 [43]. Data on model selection appear in Multimedia Appendix 1. DPC: daily percent change; VET: Veterans. *The DPC is significantly different from 0 at an α level of .05.

**Figure 3:**
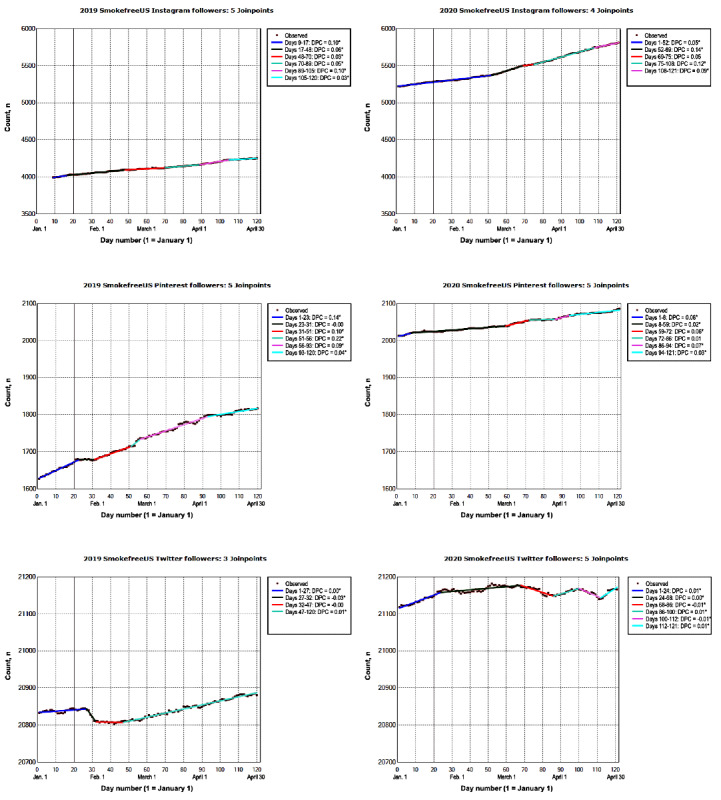
The number of SmokeFreeUS Instagram, Pinterest, and Twitter followers from January to April 2019 and 2020. The vertical line represents January 20. On this day in 2020, the first laboratory-confirmed COVID-19 case was identified in the United States. This was reported to the Centers for Disease Control and Prevention on January 22, 2020 [43]. Data on the number of daily SmokeFree Instagram followers were missing for the first 8 days in January 2019. Data on model selection appear in Multimedia Appendix 1. DPC: daily percent change. *The DPC is significantly different from 0 at an α level of .05.

In 2020, SmokeFree social media accounts had an average number of daily followers that ranged from 605 (SmokeFree Veterans Facebook) to 31,623 (SmokeFree Women Facebook; Figures 2 and 3). Social media accounts exhibited a steady but small increase in the number of daily followers (<0.1%); the ADPCs ranged from a low of 0.002% (SmokeFree US Twitter: 95% CI 0.001% to 0.002%) to a high of 0.08% (SmokeFreeUS Instagram: 95% CI 0.08% to 0.09%). In 2019, the average number of daily followers on SmokeFree social media accounts ranged from 549 (SmokeFree Veterans Facebook) to 28,244 (SmokeFree Women Facebook). SmokeFree social media accounts exhibited a steady but small increase in the number of daily followers (≤0.1%) from January to April 2019; the ADPCs ranged from a low of 0.002% (SmokeFreeUS Twitter 95% CI 0.001% to 0.002%) to a high of 0.09% (SmokeFreeUS Pinterest: 95% CI 0.08% to 0.09%).

### Smoking Cessation Interventions

#### Mobile Apps

**Figure 4:**
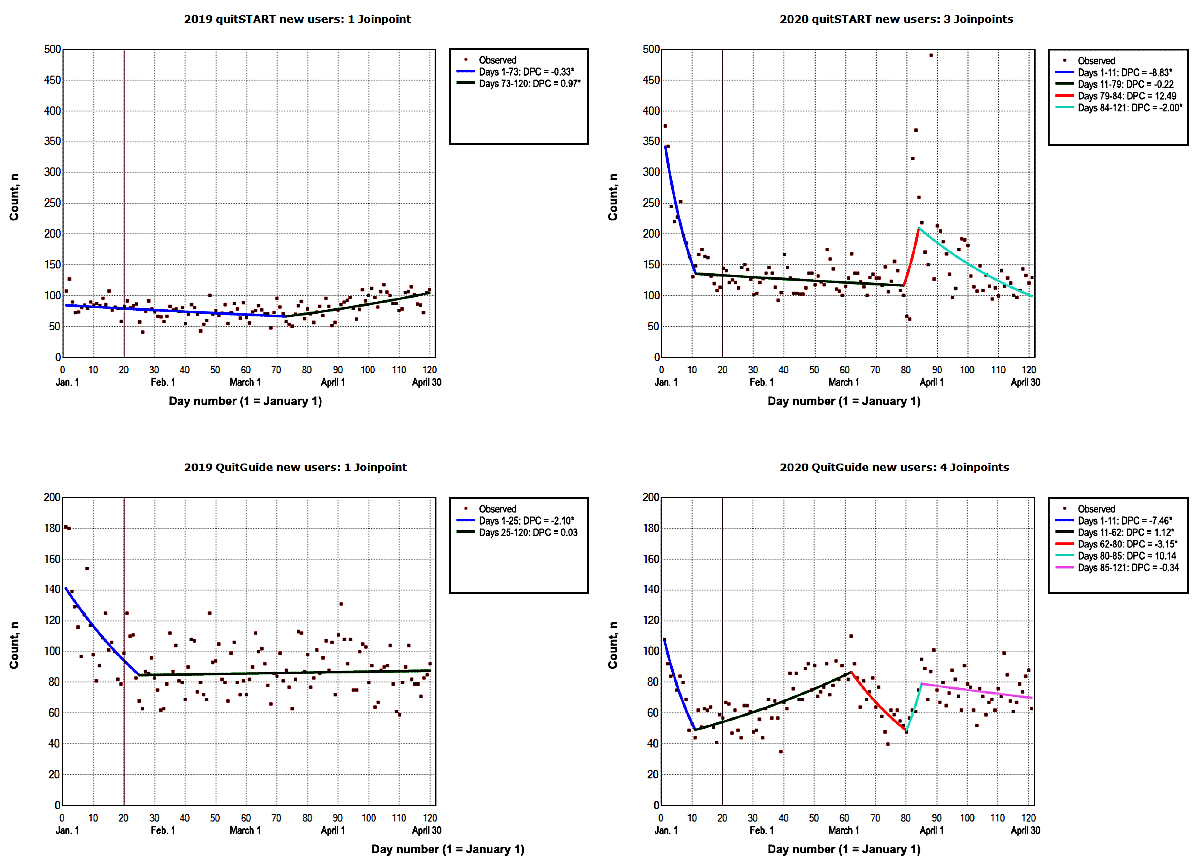
The number of new quitSTART and QuitGuide subscribers from January to April 2019 and 2020. The vertical line represents January 20. On this day in 2020, the first laboratory-confirmed COVID-19 case was identified in the United States. This was reported to the Centers for Disease Control and Prevention on January 22, 2020 [43]. Data on model selection appear in Multimedia Appendix 1. DPC: daily percent change. *The DPC is significantly different from 0 at an α level of .05.

In 2020, the number of new daily subscribers to quitSTART ranged from 62 (day 81) to 491 (day 88), and the number of new daily subscribers to QuitGuide ranged from 35 (day 39) to 110 (day 62; Figure 4). The number of new daily subscribers decreased across all intervention platforms until day 11 (quitSTART: DPC=−8.82%; 95% CI −12.82% to −4.64%; QuitGuide: DPC=−7.45%; 95% CI −10.64% to −4.15%; Table 1). The number of daily new subscribers to quitSTART did not significantly change from day 11 to day 84 (days 11-79: *P*=.08; days 79-84: *P*=.20). However, this number decreased by a DPC of −2.00% (95% CI −2.61% to −1.38%) from day 84 to day 121. For QuitGuide, the number of new daily subscribers increased by a DPC of 1.11% (95% CI 0.80% to 1.43%) from day 11 to day 62. Afterward, this number decreased by a DPC of −3.14% (95% CI −4.65% to −1.60%). From January to April 2020, the overall number of new daily subscribers to quitSTART trended downward (ADPC=−1.02%; 95% CI −1.88% to −0.15%), whereas the overall trend for QuitGuide (ADPC=−0.35%; 95% CI −1.06% to 0.37%) was not significant (*P*=.33; Table 2). In 2019, the number of new daily subscribers to quitSTART decreased by a DPC of −0.33% (95% CI −0.54% to −0.12%) between days 1 and 73, and the number of new subscribers to QuitGuide decreased by a DPC of −2.10% (95% CI −3.10% to −1.08) from day 1 to day 25. This was followed by a period of no significant changes in the number of daily subscribers to QuitGuide (*P*=.60). The overall number of new daily subscribers to QuitGuide trended downward from January to April 2019 (ADPC=−0.39%; 95% CI −0.62% to −0.17%), whereas the overall trend for quitSTART (ADPC=0.17%; 95% CI −0.01% to 0.37%) was not significant (*P*=.07).

#### Text Messaging

**Figure 5:**
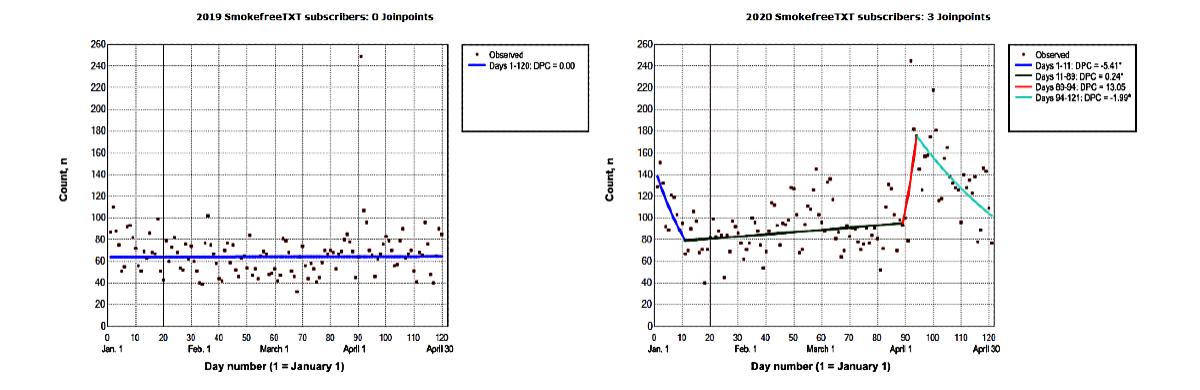
The number of new SmokeFreeTXT subscribers from January to April 2019 and 2020. The vertical line represents January 20. On this day in 2020, the first laboratory-confirmed COVID-19 case was identified in the United States. This was reported to the Centers for Disease Control and Prevention on January 22, 2020 [43]. Data on model selection appear in Multimedia Appendix 1. DPC: daily percent change. *The DPC is significantly different from 0 at an α level of .05.

In 2020, the number of new daily subscribers to SmokeFreeTXT ranged from 40 (day 18) to 245 (day 92; Figure 5). The number of new daily subscribers to SmokeFreeTXT decreased by a DPC of −5.41% (95% CI −10.05% to −0.52%) until day 11 (Table 1). Additionally, the number of new daily subscribers to SmokeFreeTXT increased by a DPC of 0.23% (95% CI 0.004% to 0.47%) between days 11 and 89. This was followed by a period of no significant changes from day 89 to day 94 (*P*=.23) and a decrease in the number of new subscribers (DPC=−1.99%; 95% CI −3.09%, to −0.87%) from day 94 to day 121. From January to April 2020, the overall number of new daily subscribers to SmokeFreeTXT (ADPC=−0.25%; 95% CI −1.22% to 0.73%) was not significant (*P*=.61; Table 2). In 2019, the overall number of new daily subscribers to SmokeFreeTXT (ADPC=0.004%; 95% CI −0.14% to 0.15%) was not significant (*P*=.95).

## Discussion

### Principal Findings

The COVID-19 pandemic is a natural event that has provided researchers with the opportunity to compare prepandemic and postpandemic digital platform traffic levels to understand the nature and magnitude of changes (if any) in the public’s health perceptions and behaviors. This study showed that overall traffic trends on the SmokeFree website and adult-focused smoking cessation interventions fluctuated from January to April 2020. Although significant increases in the number of daily visitors and subscribers to these platforms were observed, they were eventually followed by declines in traffic. Contrastingly, there were no significant peaks in the 2019 traffic trends on the SmokeFree website and the adult-focused cessation interventions, suggesting that these trends were qualitatively different from those observed in 2020. The teen-focused smoking cessation intervention (ie, quitSTART) exhibited an overall downward trend in the number of new daily subscribers over the 4-month study period in 2020. The number of SmokeFree social media account followers modestly and steadily increased over the 4-month study periods in 2019 and 2020. Peaks in SmokeFree digital platform traffic in 2020 could be attributed to an increased interest in smoking cessation among smokers during the COVID-19 pandemic.

Digital platforms are beneficial for public health surveillance [44]. Previous research has shown that search queries, website traffic, and social media use reflect disease-related information-seeking behaviors, which can serve as timely indicators of infectious disease outbreaks (ie, during times when these behaviors deviate from normal patterns) [26,44-46]. In this study, the number of new daily visitors on the SmokeFree website increased significantly starting in early February 2020 (days 39-44: *P*=.006), and although traffic levels slightly decreased afterward, they remained higher than those of January. The trends observed in 2020 were qualitatively different from those observed in 2019; the number of new daily visitors on the website modestly increased throughout the 4-month study period in 2019. Elevated website traffic in 2020 can be viewed as a proxy indicator of interest in smoking cessation information, which is driven by several factors such as media coverage of the pandemic [47], smokers’ perceived risk of SARS-CoV-2 infection [16], and calls from public health professionals to quit smoking as a preventive measure against SARS-CoV-2 infection [23,48]. Previously published literature has reported on dynamic changes in media coverage, information seeking behaviors, and perceived risk over the course of an epidemic and their importance in the adoption of precautionary behaviors and the acceptance of vaccinations [49,50].

The number of new daily subscriptions to SmokeFree cessation interventions generally peaked in early January. This is consistent with self-initiated behavioral changes that are triggered by naturally occurring events, such as New Year’s Day [51,52]. After an initial decline from January levels, the number of new subscriptions to the adult-focused interventions QuitGuide and SmokeFreeTXT peaked and decreased throughout the remainder of the study period in 2020. This is consistent with previous research on people’s precautionary behaviors, which faded over the course of the H1N1 epidemic [49]. Conversely, in 2019, the number of new subscriptions plateaued after an initial decline from January levels or remained steady throughout the study period. Although studies have suggested that the use of remote smoking cessation support services has increased during the COVID-19 pandemic [25], the fluctuations we observed in 2020 trends data could be attributed to several factors. First, evidence that links smoking to SARS-CoV-2 infection has evolved over time; several studies have suggested that nicotine is a protective/therapeutic factor against SARS-CoV-2 infection [53,54]. This prompted health organizations to provide cautionary statements [23]. Other studies have also provided evidence that smoking is a risk factor for SARS-CoV-2 infection [8-12]. Therefore, evolving evidence might have resulted in seemingly mixed or inconsistent public health messages at certain times. Second, lockdown measures and pandemic-associated economic hardships could have contributed to sustained smoking behaviors [55], which could have been reflected by the fluctuating cessation intervention uptake patterns that were observed in this study. Confinement-related psychological effects and stressors, which have been shown to increase in severity during previous epidemics and quarantines [56-58], are associated with smoking behaviors [59]. Indeed, research has shown that posttraumatic depression and stress following natural disaster exposure (eg, hurricanes) indirectly result in smoking relapse [60]. With regard to COVID-19, studies have documented a desire to initiate tobacco use or relapse among nonsmokers and former smokers [61]. Similar self-reported increases in tobacco use have been documented in 30%-40% of e-cigarette users and cigar smokers during the COVID-19 pandemic [62,63].

The number of new daily subscriptions to the teen-focused SFGI intervention (ie, quitStart) exhibited an overall downward trend over the 2020 study period. Although there was a peak in the number of subscribers in mid- to late March, this peak was not statistically significant (days 79-84: *P*=.20). These results could be attributed to two factors. First, compared to adults, children aged <18 years are less susceptible to severe complications from COVID-19, including hospitalization and death [64-66]. This information could have resulted in the generation of reassuring reports (or lack thereof) on COVID-19 effects in youth. Reports of multisystem inflammatory syndrome in pediatric patients with COVID-19 emerged in March and April [67]. Any effects that these reports would have on traffic in digital smoking cessation platforms would have occurred beyond the January to April time frame of this study. Second, electronic vaping products are the most used products among high school students [68]. Accordingly, traffic on digital platforms for cigarette users might not be the best metric for analyzing interest in quit attempts among youth during the COVID-19 pandemic. Social media accounts can promote the web-based and non–web-based use of smoking cessation resources [69]. However, the number of SFGI social media followers was qualitatively similar across 2019 and 2020. Additional research is needed to understand the content of SFGI social media accounts during the pandemic. Furthermore, research is needed to understand people’s web-based activities during the pandemic, including changes in social media following to induce behavioral change [70].

It should be noted that discrepancies in the timing and magnitude of increases and decreases in traffic on SmokeFree digital platforms could be attributed to several factors—mainly the medium of the platform, the aim of the public health effort, and the target audience. However, despite the modest increases and decreases in SmokeFree traffic, these changes are meaningful due to the reach of SmokeFree platforms [35]. It is well documented that the impact of public efforts (eg, interventions) are a product of reach and effectiveness [71]. Therefore, a mere 1% increase is meaningful if it represents hundreds or thousands of SmokeFree platform users. Furthermore, disseminating SmokeFree messages on multiple channels increases these messages’ visibility and repitition, thereby increasing the likelihood of reaching a wide audience and providing higly effective messages that induce positive behavioral changes [72]. Based on findings of this study, coordinated smoking cessation campaigns, especially those for youth, should emphasize the importance of smoking cessation and raise awareness of digital smoking cessation platforms to capitalize on people’s heightened interest during the pandemic. Public health professionals should also adapt pandemic-relevant messages and strategies instead of delivering generic smoking cessation interventions.

This study has several limitations. The platforms that were examined in this study are not representative of all digital smoking cessation resources (eg, BecomeAnEx) [73] or other resources that comply with physical distancing measures (eg, quitline) [74] and are available to smokers in the United States. Furthermore, the platforms examined in this study only target cigarette smokers, whereas resources available to other tobacco or nicotine users were not included (eg, text-to-quit vaping services) [75]. Future research should examine traffic on alternative tobacco or nicotine product cessation platforms (eg, e-cigarette use among teens and young adults) and surveil nontraditional sources (eg, pharmaceutical sales of smoking cessation aids and tobacco products sales). Such research will complement our data and provide a comprehensive picture of tobacco use during the COVID-19 pandemic [76]. Additionally, website traffic, social media followers, and intervention subscriptions are not indicative of successful quit attempts. Future research should examine smoking abstinence, reductions in the number of cigarettes smoked, and product switching (eg, switching from cigarettes to e-cigarettes) among users of SFGI digital platforms during the pandemic, as evidence on people’s motivation to quit and quitting success rates during the pandemic is mixed [62,77,78]. Traffic on SFGI platforms reflects the number of smokers who are self-motivated to quit and have access to computers/mobile phones with broadband, data, and text messaging plans. Therefore, plans for waiving smokers’ fees or providing data/text messaging plans to smokers during their quit journey should be implemented, so that smokers who might lack resources can access smoking cessation digital platforms. Furthermore, research is needed to understand the racial/ethnic and socioeconomic profiles of SFGI users. Such data were not available for analysis in this study (eg, there is evidence of increased COVID-19 exposure rates and severity risk among Black and Hispanic people) [79]. Of note, several factors that could affect traffic on SmokeFree digital platforms were not considered in this study (eg, activities for promoting SmokeFree platforms, the volume of media reports on COVID-19 and smoking, etc). This study examined prepandemic and postpandemic traffic on SmokeFree platforms over 4 months. Future research should extend the time and geographic boundaries of our study to identify smoking cessation digital platform traffic trends that occur beyond these initial 4 months in countries other than the United States.

### Conclusion

This study characterized traffic trends on SFGI digital platforms from January to April 2019 and 2020. Traffic on the SmokeFree website and adult-focused interventions increased in mid-January and February 2020, whereas traffic on the teen-focused intervention exhibited an overall downward trend. Comparable trends were not observed in 2019. The number of social media followers was similar across the 2019 and 2020 study periods. The 2020 traffic trends on the SFGI website and intervention platforms reflected opposing dynamics in the relationship between people’s interest in smoking cessation as a preventive measure against COVID-19 and evolving evidence on the risk profiles of patients with COVID-19 who develop adverse outcomes, the link between smoking and COVID-19–related complications, and sustained/increased smoking due to pandemic-related stressors.

## Appendix

Multimedia Appendix 1 Results of the joinpoint regression analysis.

## Abbreviations

ADPC: average daily percentage change
DPC: daily percent change
SFGI: SmokeFree.gov initiative
