# Supplementary material for: Insights From the SmokeFree.gov Initiative Regarding the Use of Smoking Cessation Digital Platforms During the COVID-19 Pandemic: Cross-sectional Trends Analysis Study
Source: J Med Internet Res. 2021 Mar 22;23(3):e24593. doi: 10.2196/24593 (PMC7986806; doi:10.2196/24593)
Supplement: Multimedia Appendix 1 [file jmir_v23i3e24593_app1.docx]

## **Multimedia Appendix 1**

Supplementary table 1: Joinpoint model selection

|  | **2019** | | | | | | **2020** | | | | | |
| --- | --- | --- | --- | --- | --- | --- | --- | --- | --- | --- | --- | --- |
|  | Model 1 | Model 2 | Model 3 | Model 4 | Model 5 | Model 6 | Model 1 | Model 2 | Model 3 | Model 4 | Model 5 | Model 6 |
| SmokeFree.gov | **-3.673** | -3.635 | -3.584 | -3.513 | -3.404 | -3.312 | -2.815 | -2.880 | -3.338 | -3.436 | **-3.465** | -3.413 |
| SF VET facebook | -12.451 | -12.747 | -12.964 | -13.052 | -13.012 | **-13.201** | -11.489 | -11.659 | -12.687 | -12.951 | **-13.143** | -13.074 |
| SF Women facebook | -15.228 | -16.162 | -17.070 | -17.140 | -17.423 | **-17.505** | -13.331 | -14.844 | -16.207 | -16.341 | -16.763 | **-16.765** |
| SF US facebook | -13.920 | -14.635 | -15.840 | -15.989 | -16.030 | **-16.087** | -14.073 | -14.176 | -15.100 | -15.376 | -15.628 | **-15.755** |
| SF US Instagram | -12.260 | -12.673 | -13.145 | -13.588 | -13.673 | **-13.749** | -10.490 | -13.355 | -13.382 | -13.484 | **-13.738** | -13.725 |
| SF US Pinterest | -10.907 | -11.757 | -11.743 | -12.167 | -12.241 | **-12.352** | -13.037 | -13.666 | -13.782 | -13.919 | -13.959 | **-14.187** |
| SF US Twitter | -14.155 | -16.017 | -17.010 | **-17.300** | -17.253 | -17.252 | -14.658 | -15.660 | -15.806 | -16.038 | -16.042 | **-16.518** |
| quitSTART | -3.042 | **-3.213** | -3.149 | -3.072 | -2.967 | -2.879 | -2.240 | -2.405 | -2.396 | **-2.504** | -2.465 | -2.390 |
| QuitGuide | -3.143 | **-3.298** | -3.240 | -3.165 | -3.069 | -2.971 | -2.977 | -3.034 | -3.069 | -3.138 | **-3.185** | -3.140 |
| SF TXT | **-2.476** | -2.454 | -2.430 | -2.363 | -2.265 | -2.160 | -2.453 | -2.511 | -2.538 | **-2.552** | -2.548 | -2.491 |

Cells represent Bayesian Information Criterion.

Bolded cells represent selected model.

Model #1 = 0 joinpoints, Model #2 = 1 joinpoints, Model #3 = 2 joinpoints, Model #4 = 3 joinpoints, Model #5 = 4 joinpoints, Model #6 = 5 joinpoints.
